# High-quality alternative food reduces cannibalism in the predatory mite *Amblyseius herbicolus* (Acari: Phytoseiidae)

**DOI:** 10.1007/s10493-020-00500-7

**Published:** 2020-05-18

**Authors:** Ítalo Marcossi, Morgana M. Fonseca, Paola A. F. Carbajal, André Cardoso, Angelo Pallini, Arne Janssen

**Affiliations:** 1 Department of Entomology, Federal University of Viçosa, Viçosa, Minas Gerais 36570-900 Brazil; 2 Evolutionary and Population Biology, IBED, University of Amsterdam, Science Park 904, 1098 XH Amsterdam, The Netherlands

**Keywords:** Intraspecific competition, Pollen quality, Omnivory, Biological control

## Abstract

Predatory mites of the Phytoseiidae family are important biological control agents. Many species of this family are omnivores, i.e., besides on prey, they can feed on plant resources such as nectar and pollen. It has been shown that the addition of alternative food for predators to a crop enhances biological control. However, factors such as food availability and quality can also affect interactions such as cannibalism, and thus influence biological control. We investigated the role of quality of the alternative food in the tendency of *Amblyseius herbicolus* to engage in cannibalism, a common ecological interaction in many phytoseiid mite species. Cannibalism on eggs by *A. herbicolus* was significantly reduced in the presence of high-quality food (cattail pollen) compared to egg cannibalism without alternative food, whereas this was not the case in the presence of low-quality food (cotton pollen). This suggests that cattail pollen is a high-quality alternative food, not only because it results in increased development and reproduction of predators, but also because it can minimize cannibalism.

## Introduction

Providing alternative sources of food to natural enemies has become an important strategy in biological control. It allows the introduction of the biological control agent before the pest is present in crops and enables their persistence when the target pest is scarce (McMurtry and Scriven 1966; Altieri and Letourneau 1982; Landis et al. 2000; Gurr et al. 2017). Different methods can be used to provide alternative foods in crops; one is the use of non-crop plants that provide food or prey to natural enemies (Ramakers and Voet 1995; Frank 2010; Amaral et al. 2013; van Rijn et al. 2013; Avery et al. 2014; Kumar et al. 2015; Fonseca et al. 2017). Another way is to directly supply pollen or other food sources on the crop plant (van Rijn et al. 2002; Duso et al. 2004; González-Fernández et al. 2009; van Maanen et al. 2010; Adar et al. 2014; Duarte et al. 2015). Many predatory arthropods feed on pollen as alternative food, i.e., predatory bugs (Wong and Frank 2013), ladybugs (Amaral et al. 2013), lacewings (Venzon et al. 2006), hoverflies (van Rijn et al. 2013) and predatory mites (McMurtry and Scriven 1966; Nomikou et al. 2002; van Rijn et al. 2002).

The addition of alternative food such as pollen to a crop can enhance the efficacy of pest control because it increases in the densities of natural enemies, which subsequently causes a reduction in pest populations (van Rijn et al. 2002; Nomikou et al. 2010; Delisle et al. 2015; Lee and Zhang 2018). However, the nutritional value may differ among alternative food types. For instance, pollen morphology may affect its edibility and thus affect the reproductive success and population growth of natural enemies (Yue et al. 1994; van Rijn and Tanigoshi 1999; Goleva and Zebitz 2013). Furthermore, providing alternative food to natural enemies can directly affect the interactions with conspecifics (e.g., cannibalism) and with other biocontrol agents (e.g., intraguild predation) (Lucas et al. 1998; Shakya et al. 2009; Frank et al. 2010; Calabuig et al. 2018). Here, we investigate the effects of the quality of alternative food on the occurrence of cannibalism. Reduction of cannibalism can result in changes in the densities of natural enemies, and consequently, in changes of pest densities.

Cannibalism consists of killing and at least partially consuming conspecific individuals and has been recorded in more than 1300 animal taxa (Fox 1975; Elgar and Crespi 1992). Cannibalism significantly affects population dynamics (Persson et al. 2003; Rudolf 2007), but its occurrence, strength and effect varies within and among species (Fox 1975; Leonardsson 1991; Rudolf 2008). Cannibalism may result in population extinction, but also in persistence if juveniles and adults feed on different resources and the adult food source is scarce (van den Bosch et al. 1988). One group of natural enemies that is known for cannibalism are predatory mites (Schausberger 2003; Revynthi et al. 2018). These phytoseiids feed on arthropod prey of different families and many species can also feed on plant-provided food sources (e.g., pollen, nectar, plant exudates) (McMurtry and Croft 1997; van Rijn and Tanigoshi 1999; Nomikou et al. 2003; Croft et al. 2004). The use of pollen as food for generalist predatory mites in biological control has a long history, mainly related to mass rearing under laboratory conditions (McMurtry and Scriven 1965), but also as supplemental food in crops (Ramakers 1990; Nomikou et al. 2002; van Rijn et al. 2002; Montserrat et al. 2013; Adar et al. 2014; Lee and Zhang 2018).

The omnivorous predatory mite *Amblyseius herbicolus* (Chant) (Acari: Phytoseiidae) is a generalist predator of phytophagous mites and insects, capable of reproducing and developing when feeding on pollen alone (Reis et al. 2007; Rodríguez-Cruz et al. 2013; Duarte et al. 2015). Supplementing pepper plants with pollen resulted in higher densities of this predator and better control of broad mite populations (Duarte et al. 2015). These higher predator densities were obviously caused by the addition of food, but possibly also because of lower levels of cannibalism. However, the effect of pollen on the cannibalistic behaviour of this species has not been investigated before. The objective of the present study was therefore to assess the juvenile development and survival, and the oviposition rate of *A. herbicolus* on five pollen species as alternative foods. Furthermore, we investigated the effect of pollen quality on the tendency of this predatory mite to engage in cannibalism.

## Materials and methods

### **Rearing of*Amblyseius herbicolus***

A rearing of *A. herbicolus* was started with mites collected from tomato plants in gardens in the urban and rural areas of Prados (Minas Gerais, Brazil, 21º 03′ 0″ S, 44º 04′ 47″ W) by Cardoso (2019). The mites were reared on arenas made of PVC sheets (15 × 10 cm) on top of foam pads (h = 4 cm), which were kept in plastic trays (29 × 14 × 4 cm) filled with water. To avoid mite escapes, the edges of the arenas were wrapped in wet tissue paper, which also served as a water source (van Rijn and Tanigoshi 1999). Six small pieces of tent-shaped PVC sheet were placed randomly on the arena to serve as shelters. A small piece of cotton wool was placed below each tent as an oviposition site and cattail pollen was offered as food. These arenas were kept in a climate-controlled room (25 ± 1 ºC, 70 ± 10% RH, 12:12 L:D).

### Pollen sources

Five pollen species were tested as food source for *A. herbicolus*: palm (*Syagrus romanzoffiana* [Cham.] Glassman, Arecaceae), castor bean (*Ricinus communis* L., Euphorbiaceae), corn (*Zea mays* L., Poaceae), cattail (*Typha* sp., Typhaceae) and cotton pollen (*Gossypium hirsutum* L., Malvaceae). They were all obtained from pesticide-free and non-transgenic plants on the campus of the Federal University of Viçosa (Minas Gerais, Brazil). From palm and castor bean, flowering bunches and branches were collected, respectively. They were cut, and their stems were placed in buckets filled with water to maintain turgidity until the flowers opened. After 72 h, paper was placed under the reproductive structures to collect falling pollen, which was transferred to Petri dishes (Ø = 10 cm, h = 1 cm). Corn and cattail pollen were obtained by collecting male reproductive structures of the plants and shaking them above a Petri dish. Cotton pollen was taken from the flowers of the plants with a fine brush and was transferred to a Petri dish. All pollen were dried in an oven (50 ºC) for 24 h and subsequently transferred to plastic tubes (Ø = 1 cm, h = 5 cm) and stored in a refrigerator at 4 °C until further use.

### Experimental arenas

The experimental units used for all experiments consisted of black plastic Petri dishes (Ø = 5 cm, h = 1.5 cm) closed with transparent lids. The black color of the Petri dishes allowed better observation of the mites. A small piece of tissue paper soaked in water was placed in each arena as a source of water. All adults used in the experiments were gravid females derived from the rearing arenas, aged between 10 and 12 days after egg eclosion (5–7 days old since becoming adult). All experiments were conducted in the same climate-controlled room as above.

### Juvenile development and survival

To evaluate the suitability of different diets as a food source for phytoseiid mites, it is common to estimate the intrinsic growth rate (r_m_), performing a full life table study. However, this method requires frequent evaluations for extensive periods (Van Dinh et al. 1988). Indeed, a simpler method to compare the performance of phytoseiids among diets, including prey, is to measure the peak rate of oviposition, developmental rate and juvenile survival (Janssen and Sabelis 1992; Nomikou et al. 2001).

Newly hatched larvae of *A. herbicolus* were removed from the stock colony and each individual was placed in an experimental unit. Ample amounts of one of the five species of pollen were offered on small pieces of plastic PVC (1 cm^2^). To avoid depletion and loss of quality of the pollen, new pollen was offered daily on new pieces of plastic PVC; those of the previous day were removed to avoid contamination with fungi. Twenty replicates were carried out for each pollen species. Juvenile development and survival were monitored daily until the individuals reached adulthood or had died. Data on the effect of different species of pollen on development time and survival were analysed with Cox proportional hazards model of the ‘survival’ package (Therneau 2013) in R v.3.6.0 (R Core Team 2019).

### Oviposition

For each replicate, a single adult female of *A. herbicolus* was taken from the rearing and placed on an experimental unit as above. Oviposition was recorded daily for 5 days, but the oviposition of the first day was not included in the analysis because of the possible effect of the diet of the preceding days (Sabelis 1990). The eggs were counted daily while removing them from the arenas. The effect of pollen species on the oviposition rate was assessed with a linear mixed-effects model with treatment and time as fixed factors and individuals as a random factor to correct for repeated measures. Contrasts among treatments were assessed with the Tukey method with the package lsmeans (Lenth 2016).

### Effect of diet quality on cannibalism

Based on the experiments above, cattail pollen (*Typha* sp.) and cotton pollen (*G. hirsutum)* were selected as the best and worst diet, respectively. We subsequently investigated the effect of pollen quality on the tendency of *A. herbicolus* to engage in cannibalism. *Amblyseius herbicolus* eggs were used as the stage to be cannibalized by adult females.

Adult females were placed singly on the experimental units and were starved for 24 h before being used in the experiment to avoid possible effects of the previous diet. Then, they were placed in the Petri dishes together with six conspecific eggs (< 24 h old). Ample amounts of cattail or cotton pollen were placed in the experimental units, except for control treatment, which had no pollen. The numbers of cannibalized eggs were assessed after 1, 3, 5, 20 and 24 h using a stereoscopic microscope (Zeiss Stemi 2000-c). Cannibalized eggs were recognized by the shell from which the internal egg content was removed (adapted from Yao and Chant 1989). No eggs hatched during the experiment. Twenty replicates were carried out for each diet. Data on the effect of diet on cannibalism were analysed with a Cox mixed effects model (coxme) of the ‘survival’ package (Therneau 2015). Diets (cattail pollen, cotton pollen and no food) were analysed as fixed factors and replicate as random factor to correct for repeated measures. All analyses were performed with the statistical software R (R Core Team 2019).

## Results

### Juvenile development and survival

**Fig. 1:**
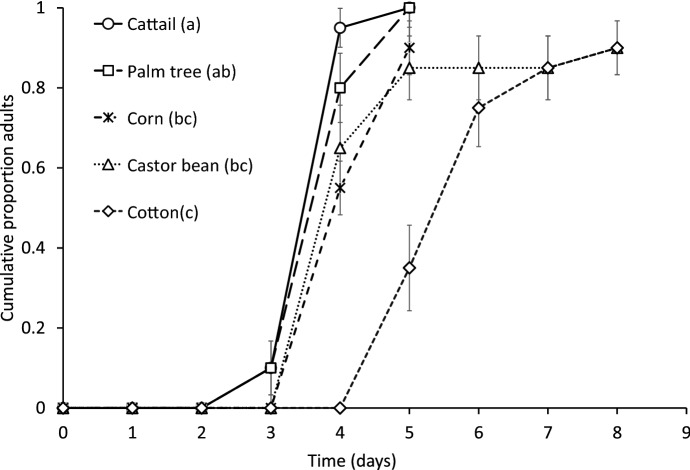
Development and survival of *Amblyseius herbicolus* juveniles fed on pollen from cattail, palm, corn, castor bean and cotton. Shown are the mean (± SE) cumulative proportions of individuals that reached adulthood as a function of time. Survival is given by the final proportion of adults. Juvenile development in treatments with different letters (see the key entries) differed significantly (contrasts after survival analysis, *P* < 0.05)

Diets of different species of pollen significantly affected the juvenile development of the predator (Fig. 1, Cox proportional hazards: likelihood ratio = 37.3, df = 4, *P* < 0.0001). Juvenile development was shorter on diets consisting of cattail pollen and palm pollen. Feeding on cotton pollen resulted in the longest juvenile development. There was no significant effect of predator diet on juvenile survival (Fig. 1, Cox proportional hazards: likelihood ratio = 6.34, df = 4, *P* = 0.17).

### Oviposition

**Fig. 2:**
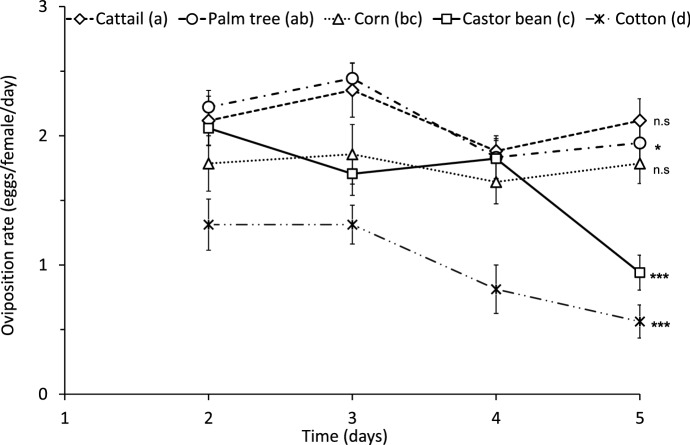
Mean (± SE) oviposition rate of *Amblyseius herbicolus* on the 2nd–5th day of feeding on a diet consisting of cattail pollen (17 replicates), palm pollen (18), corn pollen (14), castor bean pollen (17) and cotton pollen (16). Different letters following the key entries indicate significant differences among treatments (contrasts through model simplification after LME; *P* < 0.05). Asterisks indicate a significant difference in oviposition rates among days for each treatment. *0.01 < *P* < 0.05; ***P < 0.001; *n.s* not significant

There was a significant effect of pollen species on the oviposition of *A. herbicolus* (Fig. 2, LME: ${{\rm X}}_{4}^{2}$= 12.9, *P* = 0.012). Feeding on cattail and palm tree pollen resulted in the highest oviposition rates, but oviposition on palm pollen differed significantly among days (Fig. 2, cattail: *F*_1,50_ = 0.38, *P* = 0.54; palm: *F*_1,53_ = 4.49, *P* = 0.039). Oviposition on corn pollen was intermediate between palm and castor bean pollen but was not significantly different among days (Fig. 2, *F*_1,41_ = 0.06, *P* = 0.79). The oviposition rate with castor bean pollen decreased significantly among days (Fig. 2, *F*_1,50_ = 25.37, *P* < 0.001). Cotton pollen resulted in the lowest oviposition rate (Fig. 2, *F*_1,47_ = 13.75, *P* < 0.001). The sharp drop in oviposition with castor bean and cotton during the last days of evaluation may indicate longer-term negative effects of these pollens.

### Effect of diet quality on cannibalism

**Fig. 3:**
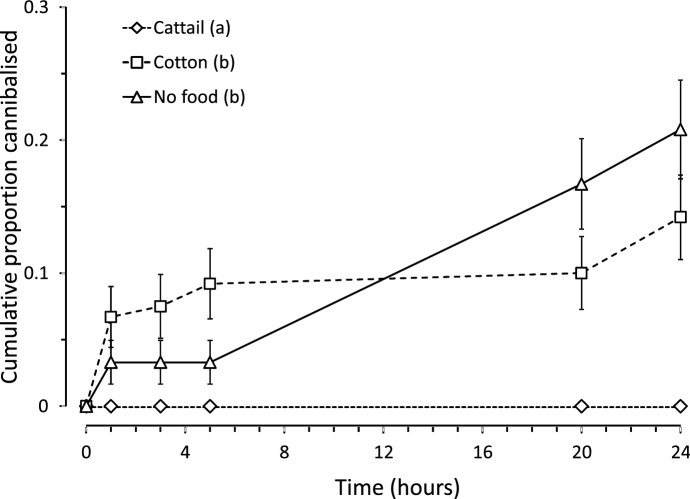
Mean (± SE) cumulative proportion of cannibalized eggs of *Amblyseius herbicolus* as a function of time in the presence of a conspecific adult predator (cannibal). Besides on the eggs, the cannibals could feed on cattail pollen (high-quality food), cotton pollen (low-quality), or had no food (control). Treatments with different letters (see the key entries) were significantly different (contrasts after a Cox mixed-effects proportional hazards model, *P* < 0.05)

No eggs were cannibalized per 24 h in the presence of cattail pollen, and less than 20% of the eggs were cannibalized in the treatment with cotton pollen and in the control (Fig. 3). Because the survival analysis requires at least some mortality in all treatments, we assumed that one egg of the treatment with cattail pollen was cannibalized at the maximum duration of the experiment (24 h). Notice that this will result in underestimating the difference among treatments. The resulting statistical analysis showed that there was a significant effect of diet quality on cannibalism (Fig. 3, Cox mixed effects: Log likelihood ratio = 17.7, df = 2, *P* < 0.001) and the cattail treatment differed significantly from the two other treatments, showing that cannibalism was affected by diet quality.

## Discussion

The predatory mite *A. herbicolus* was able to develop and reproduce when fed each of the five species of pollen under laboratory conditions, showing that they fed on all pollen species. However, the pollen clearly affected oviposition rates and juvenile development differentially; hence, they differed in quality (Figs. 1 and 2). We also show that egg cannibalism by *A. herbicolus* adults was affected by the quality of the alternative diet offered. Mites fed with cattail pollen, the superior food source, did not display cannibalistic behaviour; adult females that fed on cotton pollen, the inferior diet, and mites without food cannibalized significantly more (Fig. 3).

One of the main benefits of cannibalism is the acquisition of nutrients, especially when food availability is low (Fox 1975; van den Bosch et al. 1988). However, there is common consensus that there is no nutritional benefit of cannibalism when optimal conditions are present (Via 1999). Thus, cannibalism is expected to be more prevalent with nutritionally inferior food sources (Vangansbeke et al. 2014), in agreement with the results obtained here (Fig. 3). Vangansbeke et al. (2014) showed that females of *Amblydromalus limonicus* fed with *Typha* pollen cannibalized less than when feeding on other alternative food sources. Ferreira et al. (2008) also found that previous feeding with cattail pollen decreased cannibalism in *Iphiseius degenerans*. *Typha* sp. was also found to be a high-quality diet in other studies (van Rijn and Tanigoshi 1999; Nomikou et al. 2003; Messelink et al. 2008; Goleva and Zebitz 2013; Lee and Zhang 2018; Ajila et al. 2019). Performance of this strain of *A. herbicolus* soon after the establishment of the culture in the lab was lower than the performance found here, showing that the predators had adapted to cattail pollen as a food source.

In contrast, cannibalism was observed when cotton pollen was present, and this treatment did not differ significantly from that without food. Previous studies have also shown that cotton pollen is low-quality food for predatory mites (Elbadry and Elbenhawy 1968; Zaher and Shehata 1971). This may be due to low concentrations of some nutrients in this pollen. The absence—or reduced concentration—of specific factors in food, such as protein, vitamins or minerals, have been implicated as stimuli for cannibalism, especially related to the nitrogen content (Wolcott and Wolcott 1984). Cannibalism, then, may be an ideal strategy for N-limited predators to meet nutritional requirements when prey is scarce or when alternative foods are of low quality (Denno and Fagan 2003). Another reason for the low quality of pollen may be the presence of defensive compounds. Although concentrations of secondary metabolites in pollen are possibly too low to be harmful to predatory mites (Ranabhat et al. 2014), negative effects may occur. The sharp drop in oviposition with cotton pollen, also observed for castor bean during the last days of the experiment (Fig. 2), may indicate long-term accumulation of these secondary metabolites in the mites. Furthermore, there is possibly a relationship between the ability of mites to feed and develop on certain species of pollen with differences in mite morphology (e.g., feeding apparatus, sensory organs), physiology (digestive system) and behaviour (e.g., feeding preferences) (van Rijn and Tanigoshi 1999). These relationships require further investigation for *A. herbicolus*.

Proximate mechanisms may also explain why hunger and decreased diet quality promote cannibalism. First, hunger triggers foraging behaviour, increasing the likelihood of intraspecific contact and hence cannibalism (Charnov 1976). Second, foraging theory predicts that consumers should expand their diet during periods of hunger or low levels of food (Charnov 1976). Under this scenario, the new diet will include items previously ignored due to low energy gain and/or high acquisition costs, such as the risk of trying to cannibalize (Polis 1981). The lower nutrient content of cotton compared to cattail pollen probably caused reduced selectivity of the adult females, and hence engagement in cannibalism.

In nature, resources are often ephemeral and organisms will therefore commonly encounter situations without food, and thus have to adapt to feed on other available resources. The presence of alternative food can ensure the longer persistence of these organisms when food is scarce (Altieri and Letourneau 1982; Landis et al. 2000; Duarte et al. 2015; Gurr et al. 2017). Several papers have shown the potential of pollen as alternative food for predatory mites (van Rijn and Tanigoshi 1999; Nomikou et al. 2003; Goleva and Zebitz 2013; Montserrat et al. 2013; Duarte et al. 2015; Ajila et al. 2019) and how it can be used to improve biocontrol (van Rijn et al. 2002; Duso et al. 2004; González-Fernández et al. 2009; Nomikou et al. 2010; Delisle et al. 2015; Duarte et al. 2015). However, only a few studies have investigated whether pollen quality can affect interactions between natural enemies and thus influence biological control. For example, Pina et al. (2012) have shown that low-quality pollen resulted in satisfactory control of *Tetranychus urticae* by a combination of two phytoseiid mites because this pollen does not support high densities of the species *Euseius stipulatus*, which is the superior intraguild predator, thus reducing lethal and non-lethal interactions with the intraguild prey *Neoseiulus californicus*. Calabuig et al. (2018) found that feeding *E. stipulatus* with high-quality cattail pollen decreased larval cannibalism and intraguild predation by this mite. Other studies with different alternative foods have also shown that the quality of the resource can affect the levels of cannibalism and intraguild predation for various species of natural enemies (Snyder et al. 2000; Lucas et al. 2009). Here, we demonstrate that egg cannibalism by adults of *A. herbicolus* did not occur when cattail pollen was provided. Therefore, cattail pollen probably provided the necessary nutrients for *A. herbicolus*, thereby decreasing the engagement of this predator mite in cannibalism.

In conclusion, our study shows that providing high-quality alternative food will result in higher densities of the biocontrol agent *A. herbicolus*, not only because of increased development and reproduction, but also because the presence of pollen decreased cannibalism. Together, this may explain the higher densities of *A. herbicolus* on plants with pollen observed by Duarte et al. (2015). Factors affecting the densities of natural enemies will also indirectly determine the densities of the target pest species and therefore the efficacy of biocontrol. Thus, population-dynamical experiments on plants are needed to verify whether the addition of alternative food is beneficial for biological control, especially for natural enemies that display cannibalistic behaviour, simply because food is added, or also because it reduces cannibalism.
